# Point-of-care EEG in the pediatric emergency department: a systematic review

**DOI:** 10.1007/s00431-025-06059-y

**Published:** 2025-03-07

**Authors:** Leopold Simma, Anna Kammerl, Georgia Ramantani

**Affiliations:** 1 Emergency Department, University Children’s Hospital Zurich, Zurich, Switzerland; 2 Children’s Research Center, University Children’s Hospital Zurich, University of Zurich, Zurich, Switzerland; 3 Faculty of Medicine, University of Zurich, Zurich, Switzerland; 4 Department of Neuropediatrics, University Children’s Hospital Zurich, Zurich, Switzerland

**Keywords:** Electroencephalography, Seizures, Status epilepticus, Rapid response EEG, Reduced lead EEG, Pediatric emergency medicine, Point-of-care systems, Emergency service, Hospital

## Abstract

Central nervous system (CNS) disorders, including seizures, status epilepticus (SE), and altered mental status, constitute a significant proportion of cases presenting in the pediatric emergency department. EEG is essential for diagnosing nonconvulsive SE, but standard EEG is often unavailable due to resource constraints. Point-of-care EEG (pocEEG) has emerged as a viable alternative, offering rapid bedside assessment. This systematic review synthesizes existing data on the use of pocEEG in pediatric emergencies and highlights research gaps. A comprehensive search of PubMed, CINAHL, and EMBASE identified six studies on pediatric populations using simplified EEG montages, with cohort sizes ranging from 20 to 242 patients. The findings indicate that pocEEG is feasible in acute pediatric care, effectively aiding in the detection of nonconvulsive SE and other critical neurological conditions. The studies varied in electrode placement strategies, ranging from neonatal to subhairline montages.

*Conclusion*: Despite some implementation challenges, pocEEG has shown sufficient accuracy for clinical use. Further research should focus on optimizing EEG montages, refining interpretation, and assessing its impact on patient outcomes. This review underscores the potential of pocEEG to address critical care needs in pediatric emergency departments and calls for larger, standardized studies.

**Table:** 

**What is Known:** • *Central nervous system (CNS) disorders, such as seizures and altered mental status, are common and critical conditions encountered in pediatric emergency resuscitation bays.* • *EEG is essential for diagnosing nonconvulsive status epilepticus, but standard EEG is often unavailable in emergency departments due to logistical challenges, limited resources, and the need for specialized interpretation.*
**What is New:** • *Reduced-lead, point-of-care EEG (pocEEG) is a feasible alternative for real-time bedside CNS monitoring in pediatric emergency settings, aiding in the diagnosis of nonconvulsive status epilepticus and guiding the management of convulsive status epilepticus.* • *This systematic review highlights the feasibility and clinical potential of pocEEG in pediatric emergency departments and identifies key areas for further research, including the development of standardized pocEEG protocols and the integration of automated EEG analysis.*

**Supplementary Information:**

The online version contains supplementary material available at 10.1007/s00431-025-06059-y.

## Introduction

Central nervous system (CNS) disorders are common in pediatric emergency departments (PEDs), with many patients presenting with seizures or altered mental status, often requiring urgent attention [1, 2]. Status epilepticus (SE), including nonconvulsive SE (NCSE), are particularly concerning, since both conditions carry risks of significant morbidity and mortality if not promptly recognized and treated [3]. While convulsive SE is characterized by clear motor symptoms, NCSE is challenging to identify due to its subtle presentation [4] and requires EEG for diagnosis [5, 6]. However, traditional standard EEG is resource-intensive, time-consuming, and often unavailable outside regular working hours, posing practical challenges in PEDs. Standard EEG typically uses 19 scalp electrodes in a standardized 10–20 configuration, which requires trained technicians for setup, and neurophysiologists for interpretation. In 2018, only 27% of hospitals in the USA could perform EEG [7], and an estimated 2% of emergency departments had the capacity to conduct full-montage EEG on-site [8].

These limitations highlight the need for more accessible diagnostic solutions, such as reduced-lead EEG, to ensure timely diagnosis. In adult intensive care, reduced-lead subhairline montages have been compared to standard EEG [9, 10]. Reduced-lead EEG, such as point-of-care EEG (pocEEG), shows promise for rapid, bedside neurological assessment in PED patients [11]. Neuromonitoring via pocEEG may enable emergency medicine providers to expedite the detection and guide the treatment of SE and NCSE more efficiently. Despite this potential, only few studies have explored simplified EEG with reduced-lead montages in the PED [12, 13], leaving the full potential of pocEEG largely unexplored. This systematic review aims to compile current data on pocEEG use in the PED and identify gaps in this emerging field.

## Materials and methods

This systematic review has been registered in the International Prospective Register of Systematic Reviews (PROSPERO, CRD42022348402) on December 3, 2023, and conducted according to the Preferred Reporting Items for Systematic Review and Meta-Analysis Protocols 2020 (PRISMA) guidelines [14].

### Search strategy

We searched the Embase, PubMed, and CINAHL databases to identify studies that evaluate the use of reduced-lead EEG in PEDs. We developed our search strategy with the support of a librarian of the University Library Zurich. We combined MeSH terms and free text (Supplement 1). The literature search was first conducted in December 2023 and updated before submission. We used Covidence systematic review software (Veritas Health Innovation, Melbourne, Australia; available at: www.covidence.org). The reference lists of reviews and included studies were also screened for further suitable studies.

### Eligibility criteria

We considered original peer-reviewed articles published in English, German, Spanish, or French, without restrictions on publication date or study type. Inclusion criteria were studies on pediatric populations where reduced-lead EEG was performed in the ED as the primary intervention. Exclusion criteria were studies on adult patients, full montage EEG in the ED, and EEG used in other inpatient settings, such as operating theaters, intensive care, and sleep laboratories. We also excluded narrative reviews and gray literature (such as pre-prints, conference abstracts, and trial registries).

This review focuses on children and adolescents who underwent reduced-lead or pocEEG as a primary intervention in a PED. We compared pocEEG types, particularly electrode montage positions, and reported the clinical scenarios of their application. The primary outcomes were NCSE detection, impact on convulsive status epilepticus (CSE) management, and its role in evaluating altered mental status (AMS). Secondary outcomes were criteria for EEG use, montages, devices, and EEG interpretation.

### Data extraction

After removing duplicates, two independent reviewers (AK and LS) first screened titles and abstracts. The full text of each selected article was then assessed for eligibility by two reviewers. Discrepancies were resolved by consensus or by a third reviewer (GR). Data extracted included author, year and country of publication, age, study design, EEG montage, EEG device, measure used, and/or outcome measured.

### Assessment of risk of bias

The included studies were assessed for risk of bias using the Joanna Briggs Institute (JBI) checklist for analytical cross-sectional studies, which contains eight assessment criteria [15]. Scores ranged from 0 to 8, with higher scores indicating stronger methodological rigor. Studies scoring 7–8 were rated as high quality, studies scoring 5–6 as moderate quality, 3–4 as low quality, and 0–2 as very low quality (Supplement 2).

## Results

### Study selection

**Fig. 1:**
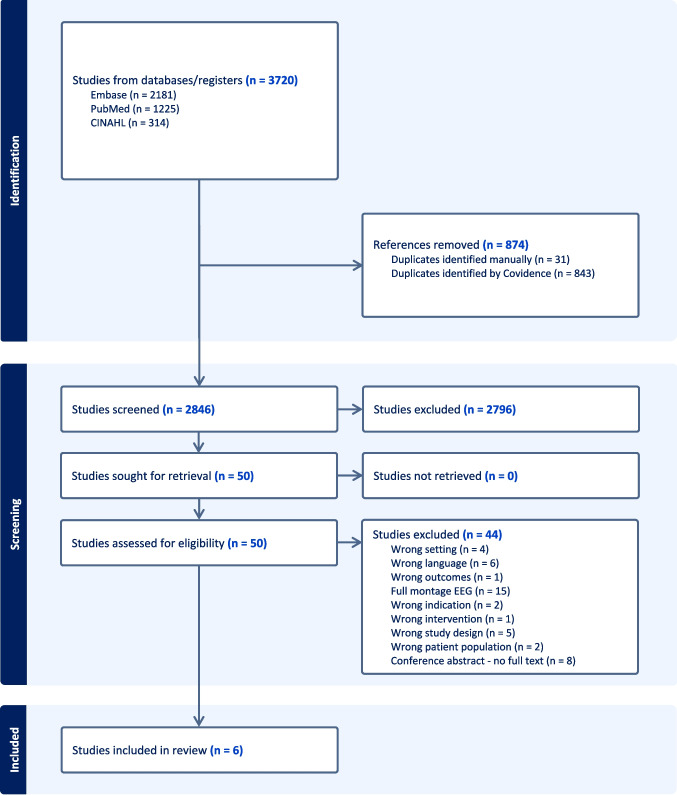
PRISMA Flowchart

Using our search strategy, we identified 3720 references. After removal of 874 duplicates, 2846 references were screened for eligibility, and 50 full-text articles were assessed (Fig. 1). Six studies met the inclusion criteria, comprising retrospective observational designs and one prospective feasibility study. These studies focused on pediatric patients with prolonged AMS and suspected nonconvulsive seizures (NCS). The studies were conducted between 2013 and 2022, with sample sizes ranging from 20 to 242 patients. The median ages of the participants ranged from 23 to 68.4 months. The rate of NCS or NCSE ranged from 4 to 17% in all patients assessed using pocEEG. Table 1 provides a summary of included studies.

**Table 1:** Summary of included studies

Author, year, country	Design	Sample size	Age	EEG type (channels, montage)	EEG device	Study period	Criteria for EEG application/inclusion criteria	EEG interpretation	Summary of outcomes and results
Yamaguchi et al., 2019; Japan [18]	R-O	242 (♂ = 124/51%)	Median: 43.9 m Age range: 0–18 y	4 channels, 9 electrodes: 2 frontal, 2 temporal, 2 occipital, 1 reference. (Fp1/Fp2, T3/T4, O1/O2, A1/A2)	Portable digital EEG system EEG-9100 (Nihon Kohden, Japan)	2016–2018	1) AMS with GCS ≤ 14 2) Suspicion of subclinical seizures *Exclusion in case of convulsions*	86% by emergency physicians (after training). 15% with neurological consultation	17% (41/242) of AMS patients assessed with pocEEG had NCS, representing 4% of all AMS cases (41/932). Patients with NCS were older and presented with more severe symptoms. No significant differences in total hospitalization duration, neurologic sequelae, or 30-day mortality compared to non-NCS patients
Nozawa et al., 2019; Japan [13]	R-O	86 (♂ = 48/56%)	Mean 5.7y (SD5.1) Median: 68.4 m Age range: 0–18 y	2 channels, 6 electrodes: 2 frontal, 2 parietal, 2 ground. (Fp1/Fp2; P3/P4)	Portable digital EEG system, COMET Lab-based EEG/PSG-Recording & Review (Grass-Telefactor, Warwick, RI, USA)	2013–2014	1) Prolonged AMS 2) Convulsions 3) Suspicion of “subclinical or subtle seizure” 4) Assessment of mental state or subclinical seizures in intubated patients under muscle-relaxants	Not reported	“persistent rhythmic waves”, indicative of seizure activity, were present in 8% (7/86) of patients; all were hospitalized and survived. “High-amplitude slow waves (< 2 Hz)”, associated with AMS and encephalopathy were observed in 12% (10/86); 8 regained consciousness, 1 died from unrelated complications. 2.8% (2/69) with normal pocEEG were later diagnosed with encephalopathy, indicating that a normal pocEEG cannot exclude underlying neurological conditions
Simma et al., 2020; Switzerland [12]	R-O	36 (♂ = 19/53%)	Median: 34 m, Age range: 0–16 y	2 channels, 5 electrodes: 2 frontal, 2 temporal, 1 reference (F1/F2; T5/T6)	Philips IntelliVue MX 550 patient monitor with EEG module (Philips Medical Systems, Germany)	2014–2017	1) Treatment monitoring 2) AMS 3) Acute focal neurological deficits 4) Encephalopathy assessment 5) Suspicion of: NCSE, paroxysmal epileptic phenomenon, dystonia, or tonic seizure	Emergency physicians (after training). Neuropediatric consultation possible	pocEEG was rated as useful by 92% of providers In 28% (10/36) of cases, pocEEG results led to therapy modification; NCSE detected in 17% (6/36) and ASM therapy monitored by pocEEG in 11% (4/36) cases
Takase et al., 2022; Japan [17]	R-O	52 (♂ = 24/46%)	Median, (IQR): 26 m, (16–48) Age range: 0–18 y	2 channels, 6 electrodes: 2 frontal, 2 parietal, 2 references (Fp1/Fp2; P3/P4)	COMET Lab-based EEG/PSG-Recording and Review system (Grass-Telefactor, Warwick, RI, USA)	2013–2017	1) Before or after BZ administration 2) Convulsions > 5 min) with GCS ≤ 13 or abnormal eye movements or involuntary movements *Exclusion in referral from other hospitals, seizure cessation with GCS* ≥ *14, or ongoing seizures upon arrival at ED*	Emergency physicians. Neurological consultation possible	36% (19/52) had abnormalities. 33% (17/52) had “slow waves (< 2 Hz)” indicative of encephalopathy, while 4% (2/52) had “rhythmic waves” indicative of seizure activity and received BZ BZ use in suspected NCSE cases decreased post-implementation of pocEEG (47% vs. 64%, p = 0.04), with reduced hospitalization rates but no significant changes in ICU admission, seizure recurrence, or final diagnoses
Stephens et al., 2023; Ireland [16]	P-F	20 (♂ = 8/40%)	Median, (IQR): 66 m (23–119) Age range: 0–16 y	2 channels, 5 electrodes: 2 central, 2 temporal, 1 reference (C3/C4, T3/4)	Nihon Kohden Neurofax (EEG-1200) machine (Nihon Kohden, Japan)	2021–2022	Patients with a Glasgow Coma Scale (GCS) < 11 or any reduction in GCS in children with a significant neurodisability at baseline	Feasibility study. No EEG review considered	Mean setup time was 41.3 min, with EEG recordings established within 60 min. 65% of recordings had < 25% artifacts, although quality declined during active airway management
Simma et al. 2023; Switzerland [19]	QI, TN	62 (♂ = 41/66%)	Median, (IQR) 40.5 m (11–83 months). Age range: 0–18 y	2 channels, 5 electrodes: 2 frontal, 2 temporal, 1 reference. (F7/F8; T5/T6)	Carescape B 450 standard mobile patient monitor (GE Healthcare, Finland) with EEG module	2021–2022	1) CSE 2) AMS 3) Suspected NCSE 4) Seizure monitoring	Emergency physicians and neuropediatric consultation	62 pocEEGs were performed during a QI project. Among the sample, 47% (29/62) patients had comorbidities. Recordings included seizure activity, NCSE and normal EEGs. Recording time ranged from 5 to 90 min

Abbreviations: *AMS* altered mental status, *BZ* benzodiazepine, *CSE* convulsive status epilepticus, *EEG* electroencephalogram, *GCS* Glasgow Coma scale, *ICU* intensive care unit, *IQR* interquartile range, *m* month, *NCS* nonconvulsive seizures, *NCSE* nonconvulsive status epilepticus, *pocEEG* point-of-care electroencephalogram, *QI* quality improvement, *TN* technical note, *R-O* retrospective, observational, *SE* status epilepticus, *y* year

### EEG systems and electrode placement

The studies used either dedicated portable EEG systems or EEG modules integrated into patient monitors with either two or four channels. Electrode placements included centrotemporal (neonatal approach) [16], frontoparietal [13, 17], frontoauriculoparietal [18], and subhairline (fronto-temporal) configurations [12, 19]. In studies including EEG interpretation, the same was mainly carried out by emergency physicians after training [17–19], while some studies involved assessments by neurologists or neurophysiologists [12]. The average time from ED arrival to EEG was 39.4 min [13] and 41.3 min, with one study reporting a mean of 20 min from ED physician assessment to EEG [18]. The application time averaged 5.4 min [13] and less than 5 min in other cases [12].

### Methodological assessment

Table 2 displays the methodological assessments: one study was rated high quality [16], four were rated moderate [12, 13, 17, 18], and one low [19], with none excluded due to quality ratings. Grading of Recommendations Assessment Development and Evaluation (GRADE) summary statements were not generated due to study heterogeneity [20], nor was a meta-analysis performed, as the studies demonstrated substantial heterogeneity in inclusion criteria, interventions, and outcomes.

**Table 2:** Quality assessment using the JBI checklist for cross sectional studies [14]

Study (autho /year)	Nozawa et al. (2019)	Yamaguchi et al. (2019)	Simma et al. (2020)	Takase et al. (2022)	Stephens et al. (2023)	Simma et al. (2023)
1. Clear inclusion criteria	Yes	Yes	Yes	Yes	Yes	Yes
2. Study subjects and setting described	Yes	Yes	Yes	Yes	Yes	Yes
3. Valid and reliable measurement of exposure	Yes	Yes	Yes	Yes	Yes	Yes
4. Objective and standard criteria for condition	Yes	Yes	Yes	Yes	Yes	N/A
5. Identification of confounding factors	No	No	No	No	Yes	No
6. Strategies to deal with confounding factors	No	No	No	No	No	No
7. Outcomes measured reliably	Yes	Yes	Yes	Yes	Yes	N/A
8. Appropriate statistical analysis	Yes	Yes	Yes	Yes	Yes	Yes
Total score	6	6	6	6	7	4
Overall quality	Moderate	Moderate	Moderate	Moderate	High	Low

## Discussion

This systematic review examined studies on reduced-lead EEG monitoring (pocEEG) in pediatric emergency settings, highlighting its feasibility and clinical utility in acute care settings. The findings suggest that integrating pocEEG into busy emergency departments is achievable. Despite setting-specific challenges, such as artifacts during active airway management, requiring robust artifact-reduction techniques and careful interpretation, the reviewed studies indicate that pocEEG recordings are generally sufficient for clinical use, even in demanding PED conditions. The review also highlighted the clinical benefits of reduced lead-EEG monitoring, especially in detecting NCS, which are challenging to identify through clinical observation alone and carry serious risks if untreated. Prompt detection via pocEEG could provide real-time information to support timely interventions, guide clinical decision-making in ambiguous or rapidly evolving cases, and potentially improve patient outcomes. However, potential limitations exist. Reduced montages may fail to detect subtle or focal seizures detectable with standard EEG, leading to false negatives. Additionally, pocEEG interpretation in a busy emergency setting carries the risk of false positives and false negatives, potentially resulting in over- or undertreatment.

The included studies varied in their inclusion criteria: some included cases of CSE [12, 16, 19], while others focused exclusively on NCSE [13, 17, 18]. Monitoring CSE with pocEEG is, however, reasonable since 1) presumably controlled CSE can still progress to NCSE [21, 22] and 2) regular exposure to monitoring of CSE may help non-expert staff recognize seizure patterns in NCSE.

Not all studies specified how EEGs were interpreted (Table 1). In most cases, non-neurologists performed the initial EEG analysis, sometimes consulting neurologists or neurophysiologists. EEG interpretation must be approached cautiously to avoid false positives and false negatives, as the complexity of EEG patterns and potential artifacts increase the risk of misinterpretation, particularly when non-neurologists are responsible for analysis. While some training programs aim to teach EEG basics to non-experts [23], only one study has shown that adult ED physicians can be trained to interpret EEGs reliably [24]. To date, no research has assessed EEG training for PED staff, nor has published data evaluated its real-world clinical application. In the future, artificial intelligence may facilitate wider access to EEG monitoring and its interpretation.

In the included studies, pocEEG detected NCS or NCSE in 4 to 17% of assessed patients [12, 13, 17–19]. Although NCS is common in critically ill children in ICUs [25] and inpatient settings [26], it remains underrecognized in EDs [27], where its incidence appears similar to that in adult populations [28].

Although standard EEG can be a valuable tool in PEDs [5, 6, 29], its limited availability and setup delays can negatively impact outcomes [30]. Reduced-lead EEG setups offer a more accessible and faster alternative for monitoring neurological activity [11]. This review identified two main approaches: dedicated EEG machines [13, 16–18] and patient monitors equipped with EEG modules [12, 19]. While both yield interpretable EEG traces, EEG modules typically have lower sampling rates [19]. Upgrading existing monitors may simplify adoption by avoiding the need for entirely new devices at a relatively low cost.

EEG montages in the reviewed studies included four-channel setups [18] for broader spatial coverage and two-channel configurations [12, 13, 17], often with subhairline electrodes for ease of application [12, 19]. Some setups, like neonatal montages, reflect specific clinical experience or context [16]. Similar reduced montages have proven useful in ICUs for critically ill patients without access to continuous EEG. For example, a commercially available EEG module with a four-channel subhairline montage showed 68% sensitivity and 98% specificity compared to standard EEG [9]. Studies have also demonstrated a high specificity and predictive value for detecting epileptiform activity [10]. Although reduced montages are inherently less sensitive than full montages, they may still be sufficient for detecting generalized EEG abnormalities while missing focal seizures [31]. Many conditions contributing to AMS, such as NCSE, typically present with generalized EEG changes, making them detectable with pocEEG. However, variations in montage highlight the need for further research on optimal electrode placement for pocEEG in EDs, although the reported electrode application by ED staff was generally quick, enabling rapid pocEEG initiation.

PocEEG has been implemented in adult EDs and reported to reliably detect NCS [32, 33]. Also, single-channel EEG has been investigated in prehospital settings [34]. Adult emergency physicians were successfully trained to recognize of basic EEG patterns to interpret EEGs [24]. Although several commercial pocEEG devices with automated EEG alerts are available for adults [11], many studies are industry-funded, raising concerns regarding bias [33, 35]. These studies support pocEEG as a rapid screening tool for NCS and NCSE, noting financial benefits such as reduced hospital stay duration, fewer transfers, and improved reimbursement, although these findings largely stem from industry-sponsored research [36]. To date, no commercial pocEEG devices have been tested specifically for pediatric use.

Cost-effectiveness considerations include setup, training, and maintenance expenses, weighed against faster diagnosis and potentially improved patient outcomes. Training emergency physicians in EEG interpretation is crucial and requires structured education along with significant resources. Key barriers to pocEEG implementation include resistance to change, accuracy concerns relative to standard EEGs, and integration challenges with existing hospital systems.

The limitations of this review include considerable variability across the included studies, with differences in procedures and methodologies that limit direct comparisons. Variability in outcome variables, measurement units, patient age ranges, and pathologies also prevented meta-analysis, as these factors likely influenced the results. The six studies on pocEEG included here mainly had moderate evidence quality and were affected by small sample sizes and inconsistent study designs. However, the benefits of pocEEG for faster diagnosis may outweigh the risks posed by technical limitations, and support its use in time-sensitive settings.

Future research on pocEEG should define its indications, quantify its impact on patient management, and assess its diagnostic reliability. Identifying the patient populations most likely to benefit and developing standardized initiation criteria would also be valuable. Comparative outcome studies or randomized trials, though challenging from an ethical perspective, could further validate findings. The accuracy of non-expert EEG interpretation in PED settings remains unstudied and warrants investigation. Additionally, research into automated EEG analysis algorithms and machine learning-based tools may improve accuracy and usability. Finally, cost-effectiveness could be assessed through longitudinal studies on neurological function, length of hospital stay, and quality of life.

## Conclusion

This systematic review highlights the potential of pocEEG in PEDs for the rapid assessment of the central nervous system. The reviewed studies demonstrate the feasibility and clinical utility of reduced-lead EEG in detecting nonconvulsive seizures and guiding patient management. Despite methodological and technical variations, pocEEG may serve as a useful tool for timely diagnosis and intervention. However, this review highlights the need for further research in this area. Future studies should focus on the impact of pocEEG use on patient care, address diagnostic uncertainties with reduced montages, investigate non-expert EEG training, and explore the integration of artificial intelligence to optimize pocEEG use in pediatric emergency settings. Advancing pocEEG technology could elevate brain monitoring to the same level of routine care given to other vital organs, ensuring that the brain receives the attention it merits as the central driver of all patient outcomes.

## Supplementary Information

Below is the link to the electronic supplementary material.

Supplementary file1 (PDF 60 KB)

Supplementary file2 (PDF 65 KB)

## Data Availability

No datasets were generated or analysed during the current study.

## Abbreviations

AMS: Altered mental status
CNS: Central nervous system
CSE: Convulsive status epilepticus
ED: Emergency department
EEG: Electroencephalogram
GRADE: Grading of recommendations assessment, development, and evaluation
ICU: Intensive care unit
JBI: Joanna Briggs Institute
NCSE: Non-convulsive status epilepticus
NCS: Non-convulsive seizures
PED: Pediatric emergency department
pocEEG: Point-of-care electroencephalogram
PRISMA: Preferred Reporting Items for Systematic Reviews and Meta-Analysis
SE: Status epilepticus
