# Supplementary material for: Point-of-care EEG in the pediatric emergency department: a systematic review
Source: Eur J Pediatr. 2025 Mar 7;184(3):231. doi: 10.1007/s00431-025-06059-y (PMC11889061; doi:10.1007/s00431-025-06059-y)
Supplement: Supplementary file 1 — Supplementary file1 (PDF 60 KB) [file 431_2025_6059_MOESM1_ESM.pdf]

Supplement 1

Full search strategy

Database: OVID EMBASE via University of Zurich Library

('juvenile'/exp OR 'childhood'/exp OR 'adolescence'/exp OR infan*:ti,ab,kw OR juvenile*:ti,ab,kw OR child*:ti,ab,kw OR adolescen*:ti,ab,kw OR teen*:ti,ab,kw OR youth*:ti,ab,kw) AND ('electroencephalogram'/exp OR eeg:ti,ab,kw OR electroencephalog*:ti,ab,kw) AND ('hospital emergency service'/exp OR 'emergency ward'/exp OR 'emergency treatment'/exp OR emergenc*:ti,ab,kw OR casualty*:ti,ab,kw)

Database: PubMed via University of Zurich Library

("Infant"[Mesh] OR "Child"[Mesh] OR "Adolescent"[Mesh] OR infan*[tiab] OR child*[tiab] OR adolescen*[tiab] OR teen*[tiab] OR youth*[tiab]) AND ("Electroencephalography"[Mesh] OR eeg[tiab] OR electroencephalog*[tiab]) AND ("Emergency Service, Hospital"[Mesh] OR "Emergency Treatment"[Mesh] OR emergenc*[tiab])

Database Cinahl via University of Zurich Library

(MH "Infants+" OR MH "Child+" OR MH "Adolescence+" OR TI infan* OR AB infan* OR TI child* OR AB child* OR TI adolescen* OR AB adolescen* OR TI teen* OR AB teen* OR TI youth* OR AB youth*) AND (MH "Electroencephalography+" OR TI eeg OR AB eeg OR TI electroencephalog* OR AB electroencephalog*) AND (MH "Emergency Service, Hospital+" OR MH "Emergency Treatment+" OR TI emergenc* OR AB emergenc*)
