# Supplementary material for: Point-of-care EEG in the pediatric emergency department: a systematic review
Source: Eur J Pediatr. 2025 Mar 7;184(3):231. doi: 10.1007/s00431-025-06059-y (PMC11889061; doi:10.1007/s00431-025-06059-y)
Supplement: Supplementary file 2 — Supplementary file2 (PDF 65 KB) [file 431_2025_6059_MOESM2_ESM.pdf]

Supplement 2 JBI Critical Appraisal Checklist for Analytical Cross-Sectional Studies.

	Yes	No	Unclear	Not applicable
1. Were the criteria for inclusion in the sample clearly defined?	□	□	□	□
2. Were the study subjects and the setting described in detail?	□	□	□	□
3. Was the exposure measured in a valid and reliable way?	□	□	□	□
4. Were objective, standard criteria used for measurement of the condition?	□	□	□	□
5. Were confounding factors identified?	□	□	□	□
6. Were strategies to deal with confounding factors stated?	□	□	□	□
7. Were the outcomes measured in a valid and reliable way?	□	□	□	□
8. Was appropriate statistical analysis used?	□	□	□	□
